# Syndecan Transmembrane Domain Specifically Regulates Downstream Signaling Events of the Transmembrane Receptor Cytoplasmic Domain

**DOI:** 10.3390/ijms22157918

**Published:** 2021-07-24

**Authors:** Jisun Hwang, Bohee Jang, Ayoung Kim, Yejin Lee, Joonha Lee, Chungho Kim, Jinmahn Kim, Kyeong Min Moon, Kyuhyung Kim, Ram Wagle, Young-Han Song, Eok-Soo Oh

**Affiliations:** 1 Department of Life Sciences, Ewha Womans University, Seoul 03760, Korea; jisunhwang@ewha.ac.kr (J.H.); bhjang@ewha.ac.kr (B.J.); aykim1209@ewhain.net (A.K.); yejinleee@ewhain.net (Y.L.); 2 Department of Life Sciences, Korea University, Seoul 02841, Korea; john725@korea.ac.kr (J.L.); chungho@korea.ac.kr (C.K.); 3 Department of Brain and Cognitive Sciences, Daegu Gyeongbuk Institute of Science and Technology (DGIST), Daegu 42988, Korea; kim.jinmahn@gmail.com (J.K.); kmmoon92@dgist.ac.kr (K.M.M.); khkim@dgist.ac.kr (K.K.); 4 Department of Biomedical Gerontology, Ilsong Institute of Life Science, Hallym University, Anyang-si 14066, Korea; ramwagle7@gmail.com (R.W.); ysong@hallym.ac.kr (Y.-H.S.)

**Keywords:** syndecan, transmembrane domain, signal transduction, PDGFR

## Abstract

Despite the known importance of the transmembrane domain (TMD) of syndecan receptors in cell adhesion and signaling, the molecular basis for syndecan TMD function remains unknown. Using in vivo invertebrate models, we found that mammalian syndecan-2 rescued both the guidance defects in *C. elegans* hermaphrodite-specific neurons and the impaired development of the midline axons of *Drosophila* caused by the loss of endogenous syndecan. These compensatory effects, however, were reduced significantly when syndecan-2 dimerization-defective TMD mutants were introduced. To further investigate the role of the TMD, we generated a chimera, 2eTPC, comprising the TMD of syndecan-2 linked to the cytoplasmic domain of platelet-derived growth factor receptor (PDGFR). This chimera exhibited SDS-resistant dimer formation that was lost in the corresponding dimerization-defective syndecan-2 TMD mutant, 2eT(GL)PC. Moreover, 2eTPC specifically enhanced Tyr 579 and Tyr 857 phosphorylation in the PDGFR cytoplasmic domain, while the TMD mutant failed to support such phosphorylation. Finally, 2eTPC, but not 2eT(GL)PC, induced phosphorylation of Src and PI3 kinase (known downstream effectors of Tyr 579 phosphorylation) and promoted Src-mediated migration of NIH3T3 cells. Taken together, these data suggest that the TMD of a syndecan-2 specifically regulates receptor cytoplasmic domain function and subsequent downstream signaling events controlling cell behavior.

## 1. Introduction

Extracellular ligands bind to the extracellular domain of cell surface membrane receptors (e.g., growth factor receptors) to initiate a transmembrane domain (TMD)-mediated receptor transition. During this process, the TMD and the cytoplasmic domain cluster to trigger the specific intracellular signaling events that regulate various cell functions, such as cell proliferation [1]. For instance, the binding of platelet-derived growth factor receptor (PDGFR) to its ligand (PDGF) leads to dimerization of the receptor and subsequent phosphorylation of the receptor’s cytoplasmic domain at numerous intracellular tyrosine residues. Phosphorylation at different tyrosine residues will induce specific interactions of the PDGFR cytoplasmic domain with intracellular adapter molecules containing Src homology domains, which will regulate downstream signal transduction [2]. Given that the PDGFR cytoplasmic domain is physically linked to its TMD, the latter must have a means to transmit external signals specific to the state of the cytoplasmic domain. Although the functions of the PDGFR cytoplasmic domain are relatively well known, it is not clear how the TMD performs its specific regulatory roles for most cell surface receptors. 

The syndecan family of transmembrane heparan-sulfate proteoglycans consists of four members: syndecan-1, -2, -3, and -4 [3,4]. As seen for other receptors, the extracellular domain of syndecan interacts with numerous extracellular ligands to induce dimerization and/or oligomerization of the TMD, which further activates intracellular signals to regulate cell adhesion and cytoskeletal reorganization [5,6,7]. The TMDs of syndecan family members contain the G*XXX*G motif, which is important for dimerization [4]. Previous experiments with a glycine mutant (Gly → Leu) in the G*XXX*G motif showed that the motif induced SDS-resistant TMD dimerization, and this tendency was important for the regulation of syndecan functions in cells [4]. Furthermore, a phenylalanine near the G*XXX*G motif of the syndecan-2 TMD was shown to participate in the strong and stable SDS-resistant dimerization of syndecan-2, which regulates syndecan-2-related functions [8,9]. The syndecan TMD therefore crucially contributes to regulating syndecan function by transferring extracellular signals into the cells. 

The binding of a ligand to the extracellular domain of the epidermal growth factor receptor (EGFR), another tyrosine kinase receptor, induces a conformational change of the TMD and subsequent EGFR dimerization [10]. Interestingly, dimerization of the EGFR TMD via an N-terminal motif stabilizes the EGFR dimer and triggers the cytoplasmic kinase domain to catalyze the phosphorylation of tyrosine residues in the cytoplasmic domain; in contrast, dimerization of the TMD via a *C*-terminal motif leads to an inactive receptor state [10,11]. This suggests that the TMD can influence the functions of the receptor’s cytoplasmic domain. Indeed, we previously showed that the TMD-mediated oligomeric status of syndecan-4 regulates the interaction of the syndecan-4 cytoplasmic domain with actinin [12]. Moreover, mutating the integrin TMD by changing Gly-708 to Asn leads to homotrimerization and altered clustering and activation [13]. Therefore, the dimerization status of a receptor’s TMD seems to generally regulate and influence the function of the cytoplasmic domain

Although it is technically difficult to monitor functional changes in the syndecan cytoplasmic domain, the PDGFR cytoplasmic domain exhibits various tyrosine phosphorylations in response to specific ligand binding. Therefore, we herein used the PDGFR cytoplasmic domain to generate various chimeras containing the syndecan TMD and applied them to investigate: (i) if the dimerization ability of the syndecan TMD is retained in the chimeric proteins and (ii) if the syndecan TMD specifically regulates the cytoplasmic domain of chimeric proteins as a signal transducer. Based on our results, we report for the first time that the syndecan TMD specifically regulates the downstream signaling events of a relevant chimeric cytoplasmic domain.

## 2. Results

### 2.1. Transmembrane Domain Regulates Syndecan-2 Functions in Model Organisms

**Figure 1:**
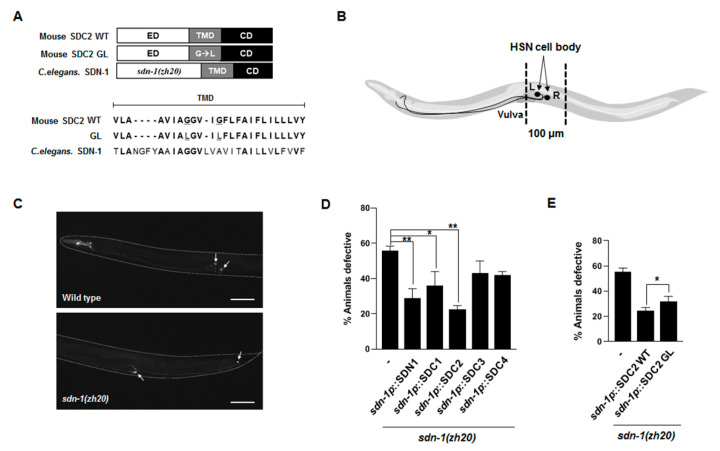
The transmembrane domain regulates syndecan-dependent HSN cell migration in *C. elegans*. (**A**) Sequence alignment of the TMD of *C. elegans* syndecan SDN-1 and mouse SDC2. Identical residues in at least two proteins are shown in bold. Mutation sites of the two SDC2 substitution mutant (GL) are underlined. (**B**) Schematic drawing of the HSN neurons in *C. elegans*. In wild-type animals, the cell bodies of two HSN neurons are located near the vulva region (L: left, R: right), and the neuronal processes run in sublateral cords to the head. In *sdn-1*(*zh20*) mutant animals, one or two cell bodies of the HSN neurons are located at the tail region. (**C**) Representative images of *tph-1*p::*gfp* expression in wild-type or *sdn-1* (*zh20*) mutant animals. Arrowheads and arrows indicate the vulva region and HSN cell bodies, respectively. Anterior is to the left. Scale bar: 100 μm. (**D**,**E**) Shown are the percentages of HSN cell migration-defective animals for the indicated genotypes. Wild-type animals exhibit 0% defect. At least two independent transgenic lines were tested. Error bars refer to the SEM; * *p* < 0.05, ** *p* < 0.001.

**Figure 2:**
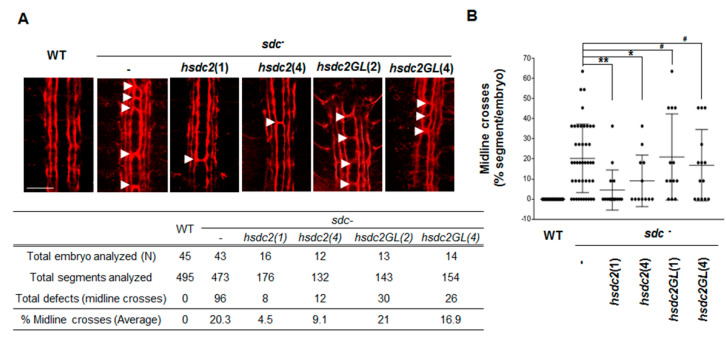
Syndecan transmembrane domain regulates development of the *Drosophila* midline axon. (**A**) Representative images from FasII-stained embryos of wild-type (WT, left column) and transgenic flies expressing wild-type or mutant *hsdc2* in the *sdc* mutant background (*sdc-*). The inner axon fascicles crossing over the ventral midline are indicated with white arrows. Scale bar = 20 µm. (**B**) Percentage of segments showing midline cross over of axon fascicles in wild-type and *sdc* mutant embryos expressing wild-type or mutant *hsdc2*. Each dot corresponds to an analyzed embryo, and at least 12 embryos were analyzed per genotype. Bars represent average ± standard deviation; * *p* < 0.05, ** *p* < 0.001, ^#^ *p* > 0.5.

Although we have shown the importance of the functional dimerization of TMD in syndecans using various cell lines [8,9], there was no direct evidence showing that this TMD-induced dimerization correlates with syndecan function in an organism. Thus, we investigated the regulatory roles of the syndecan TMD in in vivo model organisms, using *Drosophila melanogaster* and *Caenorhabditis elegans*. In contrast to mammals, which have four syndecans, each of the selected model organisms has a single syndecan homologue [14] and thus offered considerable practical advantages over a mammalian system. We first knocked down each syndecan homologue from the model organisms and then performed rescue experiments with human syndecan cDNA (Figure 1 and Figure 2). 

The *Caenorhabditis elegans* genome contains one syndecan ortholog called *sdn-1*; the predicted protein exhibits a high degree of protein sequence homology to mouse SDC2, with identities of 21, 32, and 65% in the extracellular, transmembrane, and cytoplasmic domains, respectively (Figure 1A). Previously, *sdn-1* was shown to play a role in cell migration, including that of the HSN motor neuron pair [15]. Consistent with a previous report, we found that during embryonic development about 70% of *sdn-1* mutants lacking *C. elegans* syndecan exhibited failure of the HSN neurons to migrate from the tail region to the mid-body region where the vulva is located (Figure 1B–D). To assess the degree of functional homology between mammalian syndecans and *sdn-1*, we expressed mouse syndecan cDNA under the control of the *sdn-1* promoter in *sdn-1* mutants and tested whether the HSN migration defects were restored upon expression of mouse syndecan. Indeed, mouse SDC-1, -2, -3, and -4 appeared to rescue the defects in *sdn-1* mutants, with SDC2 yielding the strongest rescue (Figure 1D). Since SDC2 expression rescued the defects in *sdn-1* mutants to nearly the same extent as expression of the *C. elegans sdn-1* cDNA, it is plausible that mammalian syndecan-2 is more functionally conserved with *C. elegans sdn-1* than other mammalian sydecans.

To next investigate the role of the TMD, we created a transgenic *sdn-1* mutant worms expressing the dimerization-defective SDC2 mutant, *sdc2GL*, which is defective in TMD-mediated dimerization due to the substitution of Gly to Leu and analyzed the HSN migration defect (Figure 1E). Although *C. elegans* expressing *sdc2GL* cDNA rescued the HSN migration defect in *sdn-1* mutants, the degree of rescue was reduced by 10% relative to that achieved by expression of the wild-type SDC2 cDNA (Figure 1E). This suggests that the TMD of mouse SDC2 is contributes to *sdn-1*-dependent HSN cell migration in *C. elegans*. 

In *Drosophila*, mutant embryos lacking *Drosophila* syndecan (*D-sdc*) exhibit axon and muscle pattern defects, and these defects are rescued by expression of human *sdc2* [16]. Therefore, we generated transgenic flies expressing wild-type *sdc2 (hscd2)* or dimerization-defective mutant *hsdc2* (*hsdc2GL*) in a *D-sdc* mutant background *(scd^-^*) (Figure 2). The wild-type embryos had three ipsilateral axon fascicles on each side of the ventral midline that stained positive for FasII, and we did not detect crossover of axon fascicles (Figure 2A, left panel and Figure 2B). In *D-sdc* mutant embryos, the innermost axon fascicles crossed over the ventral midline in 20.3% of segments (indicated by arrows in Figure 2A,B). When wild-type *hsdc2* was expressed in the *D-sdc* mutant, this defect was significantly reduced to 4.5% and 9.1% in the independent transgenic lines *hsdc2(1)* and *hsdc2(4)*, respectively (Figure 2). This confirmed that *Drosophila* syndecan can be replaced by human syndecan-2 during the development of the *Drosophila* midline axon. However, expression of dimerization-defective *hsdc2GL* increased this defect (21.0% and 16.9% in two transgenic lines) up to the level detected in *D-sdc* mutant embryos (20.3%), supporting the idea that TMD-induced dimerization contributes to regulating the function of the protein encoded by *D-sdc*. Together these data suggest that the TMD plays a critical role in regulating syndecan functions in in vivo model organisms.

### 2.2. Syndecan-2 Transmembrane Domain Is Sufficient to Induce Dimer Formation of Chimeric Proteins

**Figure 3:**
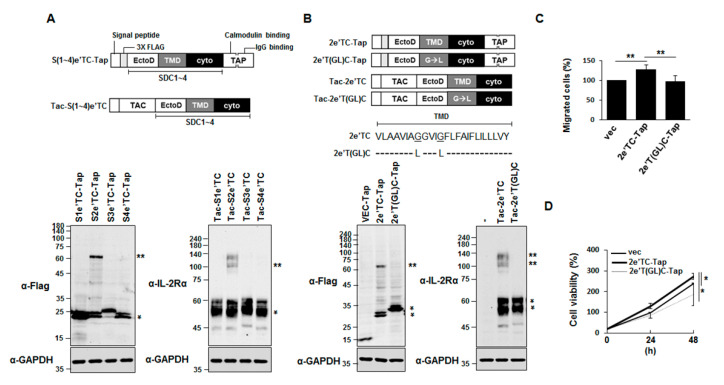
Syndecan-2 transmembrane domain induces SDS-resistant dimer formation of chimeric proteins. (**A**) Schematics of syndecan-TAP and Tac-syndecan constructs containing four amino acid residues of the extracellular domain, TMD, and cytoplasmic domain of each syndecan. The number indicates the syndecan family member (top). HEK293T cells were transiently transfected with the indicated constructs. Total cell lysates were prepared with RIPA buffer containing 2.5% SDS, separated on SDS-PAGE, and analyzed with Western blotting performed using anti-Flag or anti-IL-2Rα antibodies. Migration positions of SDS-resistant dimers (**) and monomers (*) are indicated. (**B**) HEK293T cells were transfected with 2eTC-Tap, 2eT(GL)C-Tap, Tac-2eTC, or Tac-2eT(GL)C constructs. Cells were lysed with RIPA buffer containing 2.5% SDS, separated on SDS-PAGE, and analyzed with the indicated antibodies. An antibody against GAPDH was used as a loading control. (**C**) Transwell migration assays were performed using HEK293T cells expressing syndecan-2 constructs, with 10% FBS applied as a chemoattractant in the lower chamber. Cells were allowed to migrate for 18 h and stained with 0.6% hematoxylin and 0.5% eosin. The migrated cells were counted (right). (**D**) Cells expressing the indicated syndecan-2 constructs were plated to 96-well plates and incubated for the indicated periods of time. Proliferated cells were measured by MTT assay. The data shown are representative of three independent experiments. Bars represent average ± standard deviation; * *p* < 0.05, ** *p* < 0.001.

Our group has shown that the syndecan TMD is crucial for the SDS-resistant dimerization of syndecan-2 [4], and the dimerization of a chimeric protein comprising syndecan and PDGFR was indirectly shown based on MAP kinase activation [8]. However, we lacked direct evidence showing that the syndecan TMD is sufficient to induce the dimerization of other receptor proteins in cells. Therefore, we cloned chimeric proteins in which the TMD of each syndecan was linked to either an *N*-terminal Flag-tag-conjugated Tap (Se’TC-Tap) or the extracellular domain of Tac (interleukin-2 receptor α; Tac-Se’TC; Figure 3A). Consistent with a previous report [17], Western blotting analyses performed with either an anti-Flag antibody recognizing Tap or an anti-IL-2Rα antibody recognizing Tac revealed that, among the syndecan TMD chimeras, both of the chimeric proteins containing syndecan-2 TMD (2e’TC-Tap and Tac-2e’TC) showed the strongest dimerization tendencies among the generated chimeric proteins (Figure 3A). Thus, the syndecan-2 TMD was confirmed to have the strongest dimerization tendency among the SDC family members. In addition, this SDS-resistant dimerization was not detected in dimerization-defective syndecan-2 mutants of 2e’T(GL)C-Tap and Tac-2e’T(GL)C (Figure 3B). Along with this reduced dimerization tendency, dimerization-defective syndecan-2 mutants showed much less cell migration (Figure 3C) and cell proliferation (Figure 3D). Together, these data suggest that the syndecan TMD is sufficient to induce the dimer formation of chimeric proteins.

### 2.3. Transmembrane Domain-Induced Dimerization Regulates Phosphorylation of the Cytoplasmic Domain of a Syndecan-2-PDGFR Chimeric Protein

**Figure 4:**
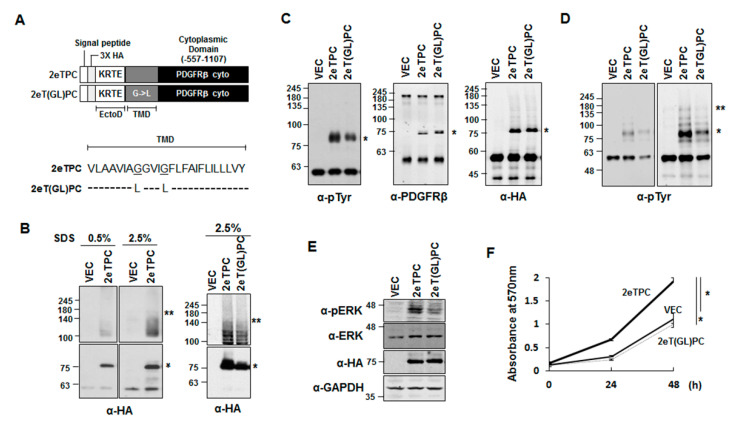
Syndecan-2 transmembrane domain induces SDS-resistant oligomerization of syndecan-2/PDGF receptor chimeras. (**A**) Schematics of the 2eTPC and 2eT(GL)PC chimeras in which four amino acid residues of the extracellular domains of rat syndecan-2 (*KRTE*) and the corresponding TMDs were linked to the cytoplasmic domain of the human PDGF (*PC*). (**B**) HEK293T cells expressing 2eTPC proteins were lysed with lysis buffer containing SDS (0.5 and 2.5%) and separated by 8 or 10% SDS-PAGE, and the proteins were detected using an anti-HA antibody. The migration positions of the SDS-resistant dimer (**) and monomer (*) are indicated. (**C**) HEK293T cells expressing the vector (vec), 2eTPC, or 2eT(GL)PC were lysed with RIPA buffer containing 2.5% SDS, and the cell lysates were diluted with RIPA buffer (final 1.25% SDS), immunoprecipitated with an anti-HA antibody, and analyzed by Western blotting with the indicated antibodies. (**D**) HEK293T cells expressing the indicated constructs were starved in 1% FBS for 24 h. Whole-cell lysates were pulled down using an anti-HA antibody and analyzed by Western blotting using an anti-pTyr antibody. (**E**) HEK293T cells expressing the indicated cDNAs were lysed using RIPA buffer, and MAPK activation was assessed using a phospho-specific antibody (pErk). (**F**) Cells were plated to 96-well plates and incubated for the indicated periods of time. Proliferated cells were measured by MTT assay. The data shown are representative of three independent experiments. Bars represent average ± standard deviation; * *p* < 0.05.

Since the syndecan TMD is sufficient to induce the dimerization of a chimeric protein (Figure 3) and we previously showed that syndecan-PDGFR chimeras containing the cytoplasmic domain of PDGF receptor can activate MAP kinase in a TMD-induced dimerization-dependent manner [4,8], we used syndecan-PDGFR chimeras together with the TMD mutants to further investigate whether TMD-induced dimerization specifically regulates syndecan signaling (Figure 4). The syndecan-PDGFR chimeras was chosen because: (i) their activation is dependent on TMD-induced dimerization; (ii) their signal transduction can be readily analyzed by MAPK activation; and (iii) this signal depends on the site-specific phosphorylation of tyrosine residues. After cells were transfected with a vector control or the chimera (2eTPC), the SDS-resistant dimer formation of 2eTPC was analyzed by Western blotting. Consistently, the syndecan-2 TMD induced the SDS-resistant dimer formation of 2eTPC even in the presence of 2.5% SDS (Figure 4B, left), whereas dimerization-defective syndecan-2 transmembrane mutants [2eT(GL)PC] showed much less of this SDS-resistant dimer formation (Figure 4B, right). When we analyzed the tyrosine phosphorylation of the chimeric proteins, we found that 2eTPC showed much higher tyrosine phosphorylation compared with the dimerization-defective mutants (Figure 4C). This was even more evident when cells were cultured in 1% FBS to reduce the background tyrosine phosphorylation (Figure 4D). These results indicate that dimerization of the TMD affected the tyrosine phosphorylation of the cytoplasmic domain of the chimeras. Since dimerization of the PDGFR cytoplasmic domain is sufficient to activate MAPK activity [18], we further analyzed MAPK activation as indicator of dimerization of these chimeras. Western blotting with anti-pErk antibodies showed that MAPK activation was much higher in 2eTPC-transfected cells than in those expressing the dimerization-defective syndecan-2 transmembrane mutants (Figure 4E). Consistent with this difference in MAPK activation, cells overexpressing 2eTPC showed a better proliferation ability (Figure 4F). Therefore, it is likely that the TMD is sufficient to induce dimerization of the cytoplasmic domain of syndecan-2/PDGF receptor chimera.

### 2.4. Transmembrane Domain-Induced Dimerization Specifically Regulates Phosphorylation on Tyr579 in the Cytoplasmic Domain of Syndecan-2-PDGF Receptor Chimera

**Figure 5:**
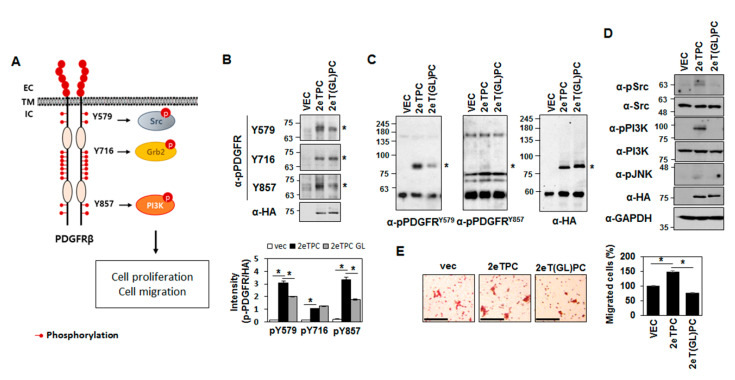
Transmembrane domain-induced dimerization specifically regulates the phosphorylation on Tyr 579 in the cytoplasmic domain of PDGF receptor. (**A**) Schematics of PDGF receptor β signaling. The site-specific tyrosine phosphorylation at the cytoplasmic domain and the relevant interacting signaling molecules are indicated. (B-E) HEK293T cells were transfected with vec, 2eTPC, and 2eT(GL)PC chimeras. (**B**) Tyrosine phosphorylation of each chimeric protein was determined by Western blotting analysis using phosphorylation-specific antibodies against p-PDGFR Y579, Y716, and Y857 (upper panel). The relative densitometric intensity was quantified (bottom panel). Migration positions of SDS-resistant monomers (*) are indicated. (**C**) Total cell lysates were immunoprecipitated with an anti-HA antibody and subjected to Western blotting with antibodies against p-PDGFR Y579, Y716, and Y857. (**D**) Whole-cell lysates were prepared and analyzed by Western blotting with the indicated antibodies. An antibody against GAPDH was used as a loading control. (**E**) HEK293T cells were used for Transwell migration assays, with 10% FBS applied as a chemoattractant in the lower chamber. Cells were allowed to migrate for 18 h and stained with 0.6% hematoxylin and 0.5% eosin (left). Scale bar = 100 µm. The migrated cells were counted (right). The data shown are representative of three independent experiments. Bars represent average ± standard deviation; * *p* < 0.05.

To further analyze the specific regulatory effect(s) of TMD-induced dimerization on the cytoplasmic domain, we investigated whether the TMD influenced the site-specific tyrosine phosphorylation of the syndecan-2-PDGFR chimera (Figure 5). The PDGFR cytoplasmic domain contains several phosphorylatable tyrosine residues, including Tyr 579, Tyr 716, and Tyr 857 [2], (Figure 5A). As expected, increased tyrosine phosphorylation of the syndecan-2-PDGFR chimera was detected in cells overexpressing the chimeric proteins (Figure 5B). Interestingly, 2eTPC-transfected cells showed increased tyrosine phosphorylation of 2eTPC, particularly at Tyr 579 and Tyr 857, but these tyrosine phosphorylations were reduced in cells expressing 2eT(GL)PC (Figure 5B). In addition, this altered tyrosine phosphorylation was more evident in chimeric proteins immunoprecipitated with HA antibody (Figure 5C). These findings indicate that TMD-induced dimerization can specifically regulate the phosphorylation of tyrosine residues in the cytoplasmic domain. The phosphorylated forms of Tyr 579, Tyr 716, and Tyr 857 have been found to interact with Src, Grb2, and PI3K, respectively [2,19,20]. Consistently, along with the increased tyrosine phosphorylation of Tyr 579, 2eTPC but not 2eT(GL)PC was associated with increased phosphorylations of Src and PI3K (Figure 5D). Furthermore, the 2eTPC-mediated increase in cell migration was reduced in cells expressing 2eT(GL)PC (Figure 5E). These results confirm that the TMD influences the phosphorylation of the cytoplasmic domain to specifically regulate the functions of the cytoplasmic domain of our syndecan-2-PDGF receptor chimera. 

**Figure 6:**
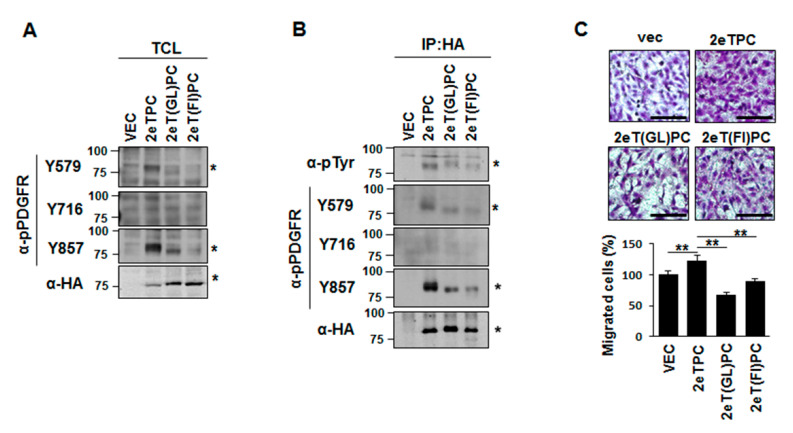
The dimerization induced by the transmembrane specifically regulates the signaling events mediated by Tyr 579 phosphorylation. (**A**) NIH3T3 cells were transfected with vec, 2eTPC, 2eT(GL)PC, and 2eT(FI)PC chimeras. Tyrosine phosphorylation in total cell lysates was determined by Western blotting analysis using phosphorylation-specific antibodies against p-PDGFR Y579, Y716, and Y857. (**B**) Total cell lysates were immunoprecipitated with an anti-HA antibody and subjected to Western blotting with phosphorylation site-specific antibodies. Migration positions of SDS-resistant monomers (*) are indicated (**C**) Transwell migration assays were performed using 10% FBS as a chemoattractant in the lower chamber. Cells were allowed to migrate for 18 h and stained with 0.6% hematoxylin and 0.5% eosin (top). Scale bar = 100 µm. The migrated cells were counted (bottom). The data shown are representative of three independent experiments. Bars represent average ± standard deviation; ** *p* < 0.001.

To further dissect the functional association of site-specific signaling events in fibroblasts, we transfected NIH3T3 cells with the syndecan-2-PDGFR chimera (Figure 6). We also used another dimerization defective syndecan-2-PDGFR chimera (2eT(FI)PC) of which Phe 167 is replaced with Ile and thus has lower dimerization ability than syndecan-2 transmembrane domain [8]. As expected, 2eTPC-transfected cells showed increased tyrosine phosphorylation of 2eTPC, but not dimerization-defective syndecan-2 mutants (e.g., 2eT(GL)PC and 2eT(FI)PC), particularly at Tyr 579 and Tyr 857 (Figure 6A,B). In addition, 2eTPC, but not the dimerization-defective syndecan-2 mutant, increased migration of NIH3T3 cells (Figure 6C), which are well-known Src-mediated cell functions. Collectively, these results indicate that TMD-induced dimerization can specifically regulate phosphorylation events in the cytoplasmic domain of the receptor to influence specific functions of the cell surface receptor.

## 3. Discussion

Although the importance of the TMDs of cell surface receptor is well recognized in transmembrane signaling, it is unclear how the TMD specifically regulates the functions of receptor proteins inside cells. In this study, we used various chimeric proteins to investigate the specific regulatory role(s) of the TMD in regulating the cell surface receptor cytoplasmic domain. First, we found that chimeric proteins containing the syndecan-2 TMD (2eTC-Tap, Tac-2eTC, and 2eTPC) formed SDS-resistant dimers through the TMD, confirming that the TMD mediated the stabilizing dimerization of this protein. Second, we observed that TMD-mediated dimerization was important for the function of syndecan in vivo. Our results revealed that syndecan-2 regulated neuronal migration in *C. elegans* (Figure 1) and the development of the *Drosophila* midline axon (Figure 2), and that dimerization-defective syndecan-2 mutants showed functional defects in both systems. The expression of mammalian syndecan-2 significantly rescued the HSN migration defect in *sdn-1* mutants, whereas the rescue of the HSN migration defect in *sdn-1* mutants was greatly reduced in *C. elegans* expressing dimerization-defective syndecan-2 mutants (Figure 1). Similarly, expression of mammalian syndecan-2 rescued the defects of ventral midline cross over in the innermost axon fascicles of *Drosophila*, whereas the dimerization-defective syndecan-2 mutants were not able to rescue these defects (Figure 2). Together, our findings suggest that proper dimerization of the TMD contributes to regulating the functions of the cell surface receptor, which is consistent with our previous results [4]. Indeed, when the syndecan-2 TMD was replaced with that of syndecan-4 abolished syndecan-2 function, suggesting that proper dimerization of the TMD contributes to regulating function of syndecan-2 [8]. A previous study showed that Phe 167 of syndecan-2 is associated with unique structural characteristics in the *C*-terminal region of the TMD [8]. However, the regulation of specific cell functions would require a unique means to specifically regulate the cytoplasmic domain, which determines downstream signal transduction within cells. Interestingly, our data revealed that phosphorylation at Tyr 579, Tyr 716, and Tyr 857 in the cytoplasmic domain of our syndecan-2-PDGF receptor chimera depended on the degree of TMD dimerization. Cells expressing 2eTPC showed more total tyrosine phosphorylation, 2eTPC tyrosine phosphorylation, ERK phosphorylation, and cell proliferation, but these effects were significantly reduced in cells expressing the dimerization-defective mutants (Figure 4, Figure 5 and Figure 6). Further, 2eTPC specifically enhanced the phosphorylation of Src and PI3 kinase, which are known downstream effectors for phosphorylated Tyr 579 in the cytoplasmic domain of PDGFR (Figure 5). Since different tyrosine phosphorylations lead to interactions with different signaling molecules in the cytosol, cell functions could be specifically regulated by dimerization of TMD. In other words, receptor-mediated intracellular signaling could depend on the dimerization status and/or structure of the TMD. This contention is consistent with our previous results regarding the hierarchy between the TMD and cytoplasmic domains in regulating the syndecan-4 cytoplasmic domain and its functions [4]. Similarly, the binding of a ligand to the G protein-coupled receptor (GPCR) induces rearrangement of the TMD helices to create a crevice at the intracellular surface of the receptor, and thus accommodate the *C*-terminus of Gα at the cytoplasmic interface to activate G protein [21,22]. Since the TMD and the cytoplasmic domain are physically linked, a structural alteration of the TMD influences the structure of the cytoplasmic domain. We speculate that TMD-mediated receptor dimerization of our chimeric protein can induce a structural change in the cytoplasmic domain to cause Tyr 579 to move the outside of the helix, where it can be easily phosphorylated by the kinase domain.

In summary, we herein reveal specific regulatory roles for the TMD of the cell surface receptor, syndecan-2. Our data show that a chimeric protein including the syndecan-2 TMD induces specific tyrosine phosphorylation of the cytoplasmic domain and its interaction with signaling molecules inside of cells, and that this depends on the dimerization status. Therefore, the TMD of a receptor can play a specific regulatory role for the cell surface receptor. Although future structural studies will be required to fully elucidate the specific regulatory mechanism of the receptor TMD, our present findings provide important new insights into the TMD-mediated signal transduction of a cell surface receptor.

## 4. Materials and Methods

### 4.1. Antibodies

Anti-PDGFRβ, anti-Erk2, anti-phospho-tyrosine (Tyr), and anti-GAPDH were purchased from Santa Cruz Biotechnology Inc. (Santa Cruz, CA, USA). Anti-HA, anti-Src, anti-phospho-Src, and anti-phospho-Erk were obtained from Cell Signaling (Beverly, MA, USA). The antibody against phospho-Tyr579 PDGFRβ were purchased from AbFrontier (Seoul, Korea), while those against phospho-Tyr716 PDGFRβ and phospho-Tyr857 PDGFRβ were purchased from Abcam (Cambridge, UK).

### 4.2. Plasmids

The chimeric protein comprising the extracellular and transmembrane domains of syndecan-2 linked with the cytoplasmic domain of human PDGFR (2eTPC) was previously described [8]. To generate a dimerization-defective syndecan-2 chimeric protein [2eT(GL)PC], a previously cloned dimerization-defective TMD of syndecan-2 [4,23] was subcloned into 2eTPC using the 5’ EcoRI and 3’ XhoI restriction enzyme sites. The sequences encoding the chimera or its point mutant were inserted into the *N*-terminal HA-tagged pcDNA3.1 expression vector (Invitrogen, Carlsbad, CA, USA). To generate Tac-syndecan constructs, the regions including the TMD and cytoplasmic domain of syndecan-1 (Ala240-Ala311), -2 (Thr132-Ala201), -3 (Ala372-Ala442), and -4 (Thr138-Ala202) were amplified by polymerase chain reaction (PCR) with 5’ NotI and 3’ XbaI restriction enzyme sites. The resulting PCR products were ligated into pcDNA3.1 encoding the extracellular domain of the interleukin-2 receptor (Tac). To generate the FLAG-tagged syndecan-TAP constructs, the PCR products were ligated into pcDNA3.1 encoding the signal sequence, 3xFLAG, and a tandem affinity purification (TAP) tag.

### 4.3. Cell Culture and Transfection

HEK293T cells and NIH3T3 cells were maintained in DMEM (Hyclone, Logan, UT) supplemented with 10% (v/v) fetal bovine serum (FBS; Gibco BRL, Grand Island, NY, USA) and gentamicin (50 µg/mL; Sigma-Aldrich, St. Louis, MO, USA). Cells were maintained at 37 °C in a humidified 5% CO_2_ atmosphere. HEK293T cells (2 × 10^5^ cells/well) were plated to 6-well plates and incubated for 24 h, and transient transfections were carried out using Vivamagic (Vivagen, Seongnam, Korea) as described in the provided manual. For stable transfection, NIH3T3 cells at 70% confluency were transfected with Vivamagic and syndecan-2 chimeric protein-expressing cells were selected with 400 µg/mL of G418.

### 4.4. Cellular Fractionation, Immunoprecipitation, and Immunoblotting

Cells were washed twice with PBS and lysed in RIPA buffer (50 mM Tris, pH 8.0, 150 mM NaCl, 1% Nonidet P-40) containing SDS (0.5 or 2.5%) to obtain whole-cell lysates. The lysis buffer contained protease inhibitors (1 µg/mL aprotinin, 1 µg/mL antipain, 5 µg/mL leupeptin, 1 µg/mL pepstatin A, and 20 µg/mL phenylmethylsulfonyl fluoride) and phosphatase inhibitors (10 µM NaF and 2 µM Na_3_VO_4_). The cell lysates were incubated for 20 min on ice and clarified by centrifugation at 13,000 rpm for 15 min at 4 °C. For membrane fractionation, washed cells were scraped off with hypotonic buffer (20 mM Tris-HCl, pH 7.5, 2 mM 2-mercaptoethanol, 5 mM EGTA, 2 mM EDTA) containing protease and phosphatase inhibitors. Lysates were incubated for 20 min on ice, and the membrane fraction was obtained by centrifugation at 13,000 rpm for 10 min at 4 °C. The pelleted membrane fraction was lysed in RIPA buffer containing protease inhibitors and sonicated. For immunoprecipitation, RIPA buffer without SDS was added to the cell lysate, and 1 mg of total proteins was incubated with anti-HA antibodies for 2 h at 4 °C. Antibody-protein complexes were precipitated with protein G-Sepharose beads (GE Healthcare, Chicago, IL, USA) and precipitated proteins were analyzed by immunoblotting. All lysates were boiled with SDS loading sample buffer for 5 min. Prepared protein samples were separated in 8 or 10% SDS-PAGE, transferred to nitrocellulose blotting membranes (GE Healthcare, Chicago, IL, USA), and probed with the indicated antibodies. The signals were detected using Odyssey (Li-Cor, Lincoln, Dearborn, NI, USA).

### 4.5. Cell Proliferation Assay

Cell proliferation was measured by a colorimetric assay using MTT([3-(4,5-dimethythiazol-2-yl) 2,5-diphenyltetrazolium bromide] (Sigma-Aldrich, St. Louis, MO, USA) according to the manufacturer’s instructions. Briefly, HEK293T cells were transfected, incubated for 24 h, harvested with TrypLE (GIBCO BRL, Grand Island, NY, USA), and seeded to 96-well plates. The cells were allowed to attach to the plate for 24 h, medium containing 0.5 mg/mL MTT was added to each well, and the cells were incubated for 1 h. The medium was then removed, and dimethyl sulfoxide (DMSO; Sigma-Aldrich, St. Louis, MO, USA) was added to each well. The mean absorbance at 570 nm was measured using a 96-well micro plate reader (Dynatech, Chantilly, VA, USA).

### 4.6. Transwell Migration Assay

The lower surface of each Transwell insert (Costar 8-μm pore size, Cambridge, MA, USA) was coated with gelatin (10 μg/mL), and the membranes were allowed to dry for 1 h at room temperature. The Transwell inserts were assembled to a 24-well plate (Costar, Cambridge, MA, USA), and the lower chamber was filled with DMEM containing 10% FBS. HEK293T cells (2 × 10^5^) were added to each upper chamber, and the plate was incubated at 37 °C in a 5% CO_2_ incubator for 24 h. Cells that migrated to the lower surface were fixed and stained with 0.6% hematoxylin and 0.5% eosin and counted.

### 4.7. Functional Analysis of hsdc2 Mutation in DROSOPHILA

All *Drosophila* strains were maintained at 25 °C. Two independent transgenic lines of *UAS-hsdc2* or *UAS-hsdc2GL* were generated by P-element-mediated germline transformation using cDNA encoding either wild-type or mutant *hsdc*2. The *elav-Gal4* and *sdc* (*sdc^23^*, designated as *sdc^-^*) mutants were obtained from Dr. Gerd Vorbrüggen (Max-Planck-Institute, München, Germany), and *w^1118^* flies were used as the wild-type. Embryo fixation and de-vitellinization were done as described previously [24]. The embryos were rehydrated in 0.1% Triton X-100 in 1X PBS (PBT) and blocked with 0.5% normal donkey serum in PBT for 30 min at room temperature. Antibody dilution and subsequent washes were done in PBT. Mouse anti-FasII (1:10; Developmental Studies Hybridoma Bank) and goat anti-mouse Alexa Fluor 568 (1:500; molecular probes) antibodies were used for staining. Images were captured using a confocal laser scanning microscope, LSM 700 (Carl Zeiss, Baden-Württemberg, Germany).

### 4.8. C. elegans Genetics and Transgenic Worms

N2 Bristol strain was used as the wild-type animals. All strains were maintained on *Escherichia coli* OP50-seeded NGM plates at 20 °C [25]. *sdn-1*(*zh20*) mutant animals and *zdIs13*(*tph-1*p::*gfp*) transgenic animals were acquired from *Caenorhabditis* Genetics Center and used in this study [15,26]. For the rescue experiments, each transgenic *C. elegans* strain was generated by microinjection of the rescue construct (50 ng/μL) with an *unc-122*p::*dsRed* marker (50 ng/μL).

### 4.9. HSN Cell Migration Assay 

Well-fed young adult hermaphrodite worms were used to observe HSN cell body migration. Each worm was placed onto a 2% agarose pad on a glass slide. Fluorescent microscopic images were taken with a Zeiss Axioplan microscope equipped with a Zeiss AxioCAM HR (Carl Zeiss, Vision GmBH, Germany). Defects in HSN migration were scored when cell bodies of the HSN neurons failed to locate within 100 μm of the vulva.

### 4.10. Statistical Analysis 

Statistical analyses were performed using the Prism 8.0 software (GraphPad Software, La Jolla, CA, USA). The difference between multiple groups of subjects was evaluated using one-way ANOVA followed by the Tukey post hoc test, or by Student’s *t* test. The SEM is indicated with error bars in each graph. A value of *p* < 0.05 was considered to indicate a statistically significant difference.

## Data Availability

Available by contacting the corresponding author.
